# Effect of Different Surface Treatments on Repair Micro-shear Bond Strength of Silica- and Zirconia-filled Composite Resins

**DOI:** 10.5681/joddd.2012.027

**Published:** 2012-12-11

**Authors:** Mohammad Joulaei, Mahmoud Bahari, Anahid Ahmadi, Siavash Savadi Oskoee

**Affiliations:** ^1^Assistant Professor, Department of Operative Dentistry, Faculty of Dentistry, Zanjan University of Medical Sciences, Zanjan, Iran; ^2^Dental and Periodontal Research Center, Tabriz Medical Sciences University, Tabriz, Iran; ^3^Assistant Professor, Department of Operative Dentistry, Faculty of Dentistry, Tabriz University of Medical Sciences, Tabriz, Iran; ^4^Department of Operative Dentistry, Faculty of Dentistry, Zanjan University of Medical Sciences, Zanjan, Iran; ^5^Associate Professor, Department of Operative Dentistry, Faculty of Dentistry, Tabriz University of Medical Sciences, Tabriz, Iran

**Keywords:** Adhesive system, composite resin, etch-and-rinse, filler, micro-shear bond strength, surface treatment, self-etch

## Abstract

**Background and aims:**

Effect of surface treatments on repair bond strength of aged composite resins might be different due to their dissimilar fillers. The aim was to evaluate the effect of different surface treatments on repair micro-shear bond strength (µSBS) of silica- (Spectrum TPH) and zirconia-filled (Filtek Z250) composite resins.

**Materials and methods:**

Twenty-seven composite resin blocks were made from each type of composite resin: Z250 and Spectrum TPH. After aging, blocks of each type were randomly divided into three groups according to surface treatments: alloy primer, silane, and only surface roughening. Subsequently, each group was further subdivided into 3 subgroups based on the adhesive system used: Single Bond, Clearfil SE Bond, and Margin Bond. Four composite resin columns were added on each block. After thermocycling, µSBStest were done at cross head speed of 0.5 mm/min. Data was analysed using multifactor ANOVA, one-way ANOVA and a post-hoc Bonferroni tests (α = 0.05).

**Results:**

Analysis of data showed that the effect of composite resin type was not significant (p > 0.05), but the effects of the type of surface treatment (p = 0.01) and the type of adhesive system (p = 0.01) were significant on repair µSBS. In addition, the cumulative effect of the composite type-surface treatment and the composite type with the type of adhesive system were not statistically significant (p > 0.05). However, the cumulative effects of the adhesive system-surface treatment (p = 0.03) and the composite type-the adhesive system-surface treatments (p = 0.002) were significant.

**Conclusion:**

Although repair µSBS values of both silica- and zirconia-filled composite resins were similar, use of different combinations of surface treatments and adhesive systems affected their repair µSBS differently.

## Introduction

One of the most frequent requirements of today's dentistry is bonding of new composite resin to an aged one. It has been estimated that half of a general practitioner’s time is spent on replacement dentistry, with the consequent high cost in time and expense.^1^ Studies have demonstrated an ideal bonding of two composite resin layers in the presence of an oxygen-inhibited layer.^2,3^ However, there is controversy over the effect of oxygen-inhibited layer on composite-composite bond strength.^4,5^ In cases with less than 24 hours' time lapse after composite curing, there are enough free and unreacted monomers with reactive vinyl groups (c = c) in the composite to copolymerize with vinyl groups of new composite resin to form a strong bond. However, after a long period from composite resin curing, these free unreacted vinyl groups tend to exit the composite, which might result in weak composite-composite repair bond strenght.^6^

According to Brosh et al^7^ the bonding of old and new composite resin in a repair case might occur by three distinct mechanisms: chemical bonding with the organic matrix, chemical bonding with exposed filler particles, and through micromechanical retention to the treated surface. As mentioned previously a strong chemical bond to resin matrix of an aged composite is questionable and in order to improve repair bond strength, different surface treatments have been used: silica coating, phosphoric acid etching followed by an adhesive,^8^ hydrofluoric acid etching, abrasion, and sandblasting.^9^ Sandblasting and etching with hydrofluoric acid are reliable methods to bond composite to porcelain.^8-13^ However, studies on the repair strength of composite resins show considerable differences and contradictions in their results. Some previous studies have failed to show a positive effect of hydrofluoric acid, sandblasting or roughening with a bur on the repair strength of composite resin,^7,14^ where most of them demonstrated a beneficial effect of such composite repair techniques.^15-19^ The variation in results might be attributed to differences in the composition of the composite resins tested in these studies as repair strengths might be influenced by the type and amount of fillers present in the composite resin.

The most commonly used filler is silica which is bonded to composite resin matrix via silane therapy. There are, however, concerns regarding the possibility of the chemical bond hydrolysis between silica fillers and resin matrix;^8,9^ therefore, zirconia-filled composite resins were introduced. Zirconia fillers are firmly retained in the resin matrix via surface microporosities. Apparently, some surface treatments used for silica-filled composite resins might not be effective in zirconia-filled ones. For example, hydrofluoric acid and silane have no reaction with zirconia fillers.^11,12^ Studies have revealed that functional organophosphate monomers can be used as primers with zirconia-filled porcelains^13,20,21^ but no study has evaluated the efficacy of these primers in zirconia-filled composite resins. Functional monomers chemically bond to metal oxides on one side and co-polymerize with resin monomers on the other.^11^

It is believed that despite similar resin matrix of the silica- and zirconia-filled composite resins, effect of surface treatments on repair bond strength would be different due to their dissimilar fillers.^23^ Therefore, the aim of present study was to evaluate the effect of different surface treatments of alloy primer, silane and only surface roughening on repair micro-shear bond strength (µSBS) of silica- and zirconia-filled composite resins.

## Materials and Methods

Silica-filled composite, TPH Spectrum (Dentsply DeTrey GmbH, Konstanz, Germany), and zirconia-filled composite, Filtek Z250 (3M ESPE, St Paul, MN, USA), were used in the present study. For each composite resin, 27 blocks measuring 15×10×1 mm were made using an aluminum split mold. Composite resin blocks were cured step-by-step by Optilux 501 (Sybron Kerr, Danbury, CT, USA) at a light intensity of 1000 mW/cm^2^ for 20 seconds in a manner in which each curing area overlapped previously cured ones to some extent. Glass slabs was placed on the bottom as well as on the top of the mold prior to curing of composite layers to provide complete adaptability to the mold walls and produce a smooth and even surface. The light intensity was frequently monitored with Optilux Radiometer (Sybron Kerr, Danbury, CT, USA) to ensure adequate polymerization of all the specimens. Composite resin blocks were immersed in distilled water at 37°C for 24 hours and then thermocycled between 5±5 and 55±5°C for 5000 cycles^23^ prior to surface roughening with a cylindrical medium-grit diamond bur (DIASWISS, Nyon, Switzerland). A new diamond bur was used for every 5 blocks. Then, the surfaces were etched using 35% phosphoric acid (Scotchbond Etchant, 3M ESPE, St Paul, MN, USA) for 20 seconds, thoroughly rinsed, and air-dried. Details of the materials used are presented in Table 1.

**Table 1:** Study materials, their manufacturer and components

Material	Manufacturer	Main Components
TPH Spectrum/Shade A1	Dentsply DeTrey, Konstanz, Germany	Bis-GMA, Bis-EMA, TEGDMA, barium aluminoborosilicate, silica
Filtek Z250/Shade A1	3M ESPE, St. Paul, MN, USA	Bis-GMA, UDMA, Bis-EMA Zirconia/silica Fillers (without silane treatment)
Scotchbond Etchant gel	3M ESPE, St. Paul, MN, USA	35% phosphoric acid, silicon dioxide
Margin Bond	Coltène/Whaledent AG, Switzerland	Bis-GMA, Bis-EMA, TEGDMA
Adper Single Bond Plus	3M ESPE, St. Paul, MN, USA	Bis-GMA, HEMA, polyalkenoiccopolymer, ethanol, photoinitiator
Clearfil SE Bond	Kuraray Medical Inc., Okayama, Japan	SE Primer: N,N-Diethanol-p-toluidine, MDP, HEMAhydrophilic dimethacrylate, DL-camphorquinone, water. SE Bond: N,N-Diethanol-p-toluidine, MDP, Bis-GMA, HEMA, hydrophobic dimethacrylate, DL camphorquinone, silanated,colloidal silica
Alloy Primer	Kuraray Medical Inc., Okayama, Japan	MDP
Porcelain Silane	Ultradent, Salt-Lake City, UT, USA	3-methacryloxy propyltrimethoxysilane, ethanol, water, acetic acid

Bis-GMA: bisphenol-glycidyl methacrylate, Bis-EMA: ethoxylatedbisphenol A glycol dimethacrylate, TEGDMA: triethylene glycol dimethacrylate, UDMA: urethane di-methacrylate, HEMA: 2-hydroxyethyl methacrylate MDP: 10-methacryloyloxydecyl dihydrogen phosphate.

### Surface Treatments for Repair of Composite Resins

Twenty-seven blocks of each composite resin type were divided into 3 groups (n = 9) according to their initial surface treatments:

1. Application of one layer of Alloy Primer (ALP) (Kuraray Medical Inc., Okayama, Japan) and air drying after 60 seconds
2. Application of one layer of coupling agent, Porcelain Silane (PS) (Ultradent, Salt-Lake City, UT, USA) and air drying after 60 seconds
3. No additional primer after surface roughening (SR)

### Application of Adhesive Systems

Each group of surface-treated composite resin blocks was further subdivided into three subgroups (n = 3) based on the adhesive system applied:

1. One layer of Margin Bond (MB) adhesive system (Coltène/Whaledent AG, Switzerland) and curing for 20 seconds
2. One layer of Clearfil SE Bond (CSE) adhesive system (Kuraray Medical Inc., Okayama, Japan) and curing for 20 seconds
3. Two layers of Single Bond (SB) adhesive system (3M ESPE, St Paul, MN, USA) and curing for 20 seconds, followed by 5 seconds of gentle air drying of each layer

### Preparation of Specimens for the µSBS Test

Iris cuts from silicone Tygon tubing (Small Parts Inc, Logansport, IN, USA) with an internal diameter of 1 mm and a height of approximately 0.5 mm were used to produce composite cylinders. On each composite resin block, four composite resin cylinders of the same type were placed with a distance of 2 mm from each other. The specimens were then thermocycled between 5±5 and 55±5°C for 1000 cycles. Then, the composite resin blocks were fixated on Universal Testing Machine (Hounsfield Test Equipment, Model H5-KS, Surray, UK) using cyanoacrylate glue (Mitreapel, Beta Chemical Ind. & Trade Inco. Co., Istanbul, Turkey); a micro-shear force with a cross-head speed of 0.5 mm/min was applied via copper wire placed around the composite cylinders.

### Statistical Analysis

Since Kolmogorov-Smirnov test revealed normal distribution of data (p > 0.05), multifactor ANOVA was used for statistical analysis. Furthermore, in cases in which the differences were statistically significant, after application of one-way ANOVA, a post-hoc Bonferroni test was used for two-by-two comparisons. Statistical significance was defined at p < 0.05.

## Results

Table 2 presents the results of multifactor ANOVA which showed that the effect of composite resin type was not significant (p > 0.05), but the effects of the type of surface treatment (p = 0.01) and the type of adhesive system (p = 0.01) were significant on repair µSBS. In addition, the cumulative effects of composite resin type with the type of surface treatment (p > 0.05) and the composite resin type with the type of the adhesive system (p > 0.05) were not statistically significant. However, the cumulative effects of the adhesive system-surface treatment (p = 0.03) and the composite resin type-adhesive system-surface treatments (p = 0.002) were significant.

**Table 2:** Results of multi-factor ANOVA for mean of repair µSBS (p < 0.05)

Source	Type III Sum of Squares	df	Mean Square	F	Sig.
Composite Type (CT)	4.134	1	4.134	0.238	0.627
Surface Treatment (ST)	150.612	2	75.306	4.328	0.015
Adhesive Systems (AS)	243.882	2	121.941	7.008	0.001
Interaction (CT-ST)	3.475	2	1.737	0.100	0.905
Interaction (CT-AS)	48.140	2	24.070	1.383	0.253
Interaction (ST-AS)	185.844	4	46.461	2.670	0.034
Interaction (CT-ST-AS)	310.114	4	77.528	4.456	0.002
Error	3288.607	189	17.400	—	—
Total	36153.446	207	—	—	—

When repairing both composite resins with ALP or SP as surface treatments there were no statistically significant differences between different adhesive systems (p > 0.05). However, with SR as surface treatment for both composite resins there were statistically significant differences between the adhesive systems (p_TPH_ < 0.001, p_Z250_= 0.03, one-way ANOVA). In this case, a Bonferroni test revealed the following: repair µSBS of TPH with SB = CSE (p > 0.05), SB = CSE > MB (p < 0.001) and repair µSBS of Z250 with SB > CSE (p = 0.02), SB = MB and CSE = MB (p > 0.05) (Table 3).

**Table 3:** Means and standard deviations (Mean ± SD) of the repair µSBS values (MPa)

Composite Resin	Surface Treatments	Adhesive Systems		
		Single Bond	Clearfil SE Bond	Margin Bond
TPH Spectrum	Alloy Primer	9.6 ± 3.8 ^aA^	11.5 ± 4.4^aA^	12.8 ± 3.9 ^Aa^
	Porcelain Silane	14.0 ± 3.2 ^bA^	14.3 ± 4.0 ^abA^	10.3 ± 4.3 ^aA^
	Surface Roughening	15.0 ± 3.6 ^bA^	15.7 ± 3.09 ^bA^	9.2 ± 2.7 ^aB^
Filtek Z250	Alloy Primer	11.1 ± 3.1 ^aAB^	13.1 ± 5.5 ^aA^	8.7 ± 3.2 ^aB^
	Porcelain Silane	13.7 ± 4.0 ^abA^	13.4 ± 4.8^aA^	11.6 ± 4.4^aA^
	Surface Roughening	15.8 ± 6.0 ^bA^	10.2 ± 3.7 ^aB^	12.2 ± 5.0^aAB^

Same lower letters indicate no significant difference in bond strength between different surface treatment groups when using similar adhesives in each composite type. Same capitals indicate no significant difference of bond strength between different adhesive groups when using similar surface treatments in each composite type.

When repairing TPH using SB or CSE adhesive systems there were statistically significant differences between surface treatments applied (p_SB_ = 0.002, p_CSE_ = 0.04, one-way ANOVA). However, for MB adhesive system there were statistically significant differences between surface treatments applied (p > 0.05). Two-by-two comparison of surface treatments for SB adhesive system revealed the following: OR = PS (p > 0.05), PS > ALP (p = 0.01) and OR > ALP (p = 0.002). Bonferroni test revealed the following: OR = PS (p > 0.05), PS = ALP (p > 0.05) and OR > ALP (p = 0.04) with the use of CSE adhesive system. In case of repairing Z250 composite with all of the adhesive systems there were no statistically significant differences between different surface treatments applied (p > 0.05) (Table 3).

## Discussion

Conventional shear and tensile tests have been criticized for using relatively large bonded surfaces, over which stress distribution is likely to be uneven in relation to the density of intrinsic faults, possibly acting as stress raisers.^24^ In this regard, the microtensile technique is considered more reliable, being able to more closely reflect the interfacial bond strength, as it offers more uniform stress distribution.^25,26^ However, a high frequency of premature failures and large standard deviation values were reported.^27^ In µSBS test, unlike microtensile technique, sectioning and trimming steps which may introduce early microcracking and pre-test failures within the specimen are avoided.^28,29^ Therefore, it is more effective and reliable for evaluating bonding efficiency of adhesive systems.^28^

Clinically, aging is a result of exposure of composite materials to the oral environment, food and beverages of all kinds, and cyclic loading over a long period of service. This aging process will result in leaching of certain components out of the composite resin, water uptake in the resin matrix and along the resin–filler interface and wear of the surface due to loss of resin matrix and filler particles.^30,31^ These changes can alter the composition of the material and will also affect the repair bond strength.^32,33^ In a recent study, Ozcan et al^15^ demonstrated that ‘aging’ using thermocycling for 5000 rounds was more efficacious than the effect of citric acid (3.5% for 1 week) and storage in boiling water for 8 hours. Therefore, in the present study the technique of thermocycling for 5000 rounds was chosen.

The present study was conducted to determine the effect of different combinations of surface treatments and adhesive systems on repair μSBS of two types of composite resins with dissimilar filler types: Filtek Z250, zirconia-filled and Spectrum TPH, silica-filled.

After aging in a humid environment, the water saturation of the composite resin was accomplished, and the monomer functional group’s radical activity diminished.^33^ Therefore, the surfaces of the aged composite resins need to be refreshed somehow. The use of an intermediate low-viscosity resin can be considered a necessary step in composite resin repair, to enhance the bond by promoting chemical coupling to the resin matrix, bonding to the exposed fillers, or micromechanical retention through monomer penetration into the matrix microcracks.^15,34^

To this end, three adhesive systems with different characteristics were used in this study: Margin Bond, an enamel bonding containing hydrophobic monomers; Single Bond, an etch-and-rinse adhesive system, containing hydrophobic monomers together with hydrophilic monomers; and Clearfil SE Bond, a two-bottle (primer + bond) self-etch adhesive system, containing functional organophosphate monomers.

On repair protocol, in all the groups surface roughening was performed with a diamond bur followed by etching with phosphoric acid prior to the application of surface primers. According to Loomans et al, besides the superficial cleaning capacity of phosphoric acid, which removes debris and grinding debris from the resin composite surface,^22,35^ phosphoric acid alone had no effect on surface roughness.^22^ Moreover, etching with phosphoric acid might also activate the reactivity between silica or zirconia surface and PS or ALP which were used as surface primers in this study.^35^ Furthermore, it should be pointed out that in the present study, for this reason and also because of strong hydrophilic nature of primer component of CSE adhesive system, which might be incompatible with mainly hydrophobic surface of composite resin, primer component of this adhesive was not applied prior to the application of bonding. We were also inspired to use MB because of mainly hydrophobic nature of composite resin surface.

In the present study, repair μSBS of TPH using SB adhesive system was higher when OR or PS were applied as surface treatment compared to that of ALP. This means that the aforementioned combination using PS as chemical primer after surface roughening does not cause any improvement in repair μSBS and use of ALP may decrease it. ALP is a metal primer containing MDP without any methylmethacrylate-based ingredient to bond with SB. When repairing TPH using CSE adhesive system repair μSBS with OR as surface treatment was similar to PS and PS was similar to ALP. However, repair μSBS with OR was significantly higher than that with ALP. These mean that in such a combination there is no need for applying chemical primers after surface roughening in order to increase repair μSBS. However, in contrast to SB, repair μSBS with PS was similar to ALP. This might be attributed to compatibility and probability of its chemical bonding to ALP primer due to the presence of MDP in their composition. Furthermore, when repairing TPH with MB adhesive system there were no significant differences between surface treatments, either.

Another finding of the present study was that no differences were found when using different combinations of adhesive systems and surface treatments in order to repair Z250 composite resin. The reason may be that since fillers comprise only a small percentage of the composite surface and the greater proportion is devoted to resin, surface treatments do not appear as efficient as in the case of porcelains.^22^ That is why adhesives such as MB, which is only composed of hydrophobic monomers without any functional groups, could wet composite surface properly.

Previously, several methods have been used in order to roughen aged composite resin surfaces. Hydrofluoric acid is corrosive and a contact poison; a meticulous application technique is needed to prevent detrimental side effects, such as acid burns and necrosis of the underlying soft tissues.^36-38^ Therefore, this material is less safe for intra-oral application. On the other hand, a major disadvantage of sandblasting is the aerosol of fine abrasive particles that will contaminate a wide area of the operatory, which might be harmful for patients and operators.^39^

However, roughening with diamond bur, used in this study, might be the simplest, the most feasible and aggressive of them. Contradictory results have been reported with the use of diamond burs for preparing composite surfaces prior to bonding.^7,33,40,41^ Nevertheless, based on the results of this study and similar to those of a study by Soderholm et al,^29^ surface roughening with diamond bur followed by acid etching with phosphoric acid and applying an adhesive system with good wetting properties seems to be the most feasible technique for dentists to use. Furthermore, applying primers such as PS or ALP, which contain functional monomers in order to react with filler particles, does not have any significant effects on the repair μSBS, regardless of the filler type.

However, it should be noted that the bond strength values do not necessarily correlate with microleakage, at least in the laboratory.^42^ Therefore, it is recommend that microleakage of repaired interfaces and SEM observations of debonded surfaces be evaluated in future. Furthermore, no data appears to exist on the correlation between bond strength of repaired composite resins and clinical performance.

## Conclusion

Although repair µSBS values of both silica- and zirconia-filled composite resins were similar, using different combinations of surface treatments and/or adhesive systems affected their repair µSBS values differently. However, based on this study and within the limitations described, surface roughening with diamond bur followed by acid etching with phosphoric acid and applying an adhesive system with good wetting properties is recommended for repairing aged composite resins, regardless of the filler type.
